# Complete mitochondrial DNA genome sequence variation of Chinese families with mutation m.3635G>A and Leber hereditary optic neuropathy

**Published:** 2012-12-30

**Authors:** Rui Bi, A-Mei Zhang, Xiaoyun Jia, Qingjiong Zhang, Yong-Gang Yao

**Affiliations:** 1 Key Laboratory of Animal Models and Human Disease Mechanisms of the Chinese Academy of Sciences & Yunnan Province, Kunming Institute of Zoology, Kunming, Yunnan, China; 2 State Key Laboratory of Ophthalmology, Zhongshan Ophthalmic Center, Sun Yat-sen University, Guangzhou, China; 3 Graduate University of the Chinese Academy of Sciences, Beijing, China

## Abstract

**Purpose:**

The majority of Leber hereditary optic neuropathy (LHON) cases are caused by one of three mitochondrial DNA (mtDNA) primary mutations (m.3460G>A, m.11778G>A, and m.14484T>C). In recent studies, we and others have shown that mutation m.3635G>A is a primary LHON mutation, particularly in Chinese. The purpose of this study was to perform a thorough analysis for the complete mtDNA genome sequence variation in Chinese patients with m.3635G>A and to identify potentially functional variants cosegregated with m.3635G>A.

**Methods:**

The complete mtDNA genomes of five Chinese patients with LHON carrying m.3635G>A were determined. A phylogenetic tree was constructed to distinguish the private and ancestral mtDNA variants in each lineage. Previously unreported variants in each mtDNA were defined with a web-based and database search. mtDNA variants that changed the structure of the membrane-spanning region of the protein were also evaluated, together with evolutionary conservation analysis, to predict their potential pathogenicity.

**Results:**

The five patients with LHON sequenced in this study belonged to haplogroups M7b4 (Le131), F1a (Le329 and Le337), B5b (Le569), and M7b6 (Le834), which suggested multiple origins of m.3635G>A. Private variants m.12811T>C, m.14063T>C, m.15237T>C, and m.9071C>T in these patients were predicted to change the structure of the membrane-spanning region of the respective proteins.

**Conclusions:**

Mutation m.3635G>A had multiple origins in Chinese patients with LHON. We also identified several potentially functional variants cosegregated with m.3635G>A.

## Introduction

Leber hereditary optic neuropathy (LHON; MIM 535000) is a typical mitochondrial genetic disease that leads to acute or sub-acute visual loss mainly in young adult men [1,2]. Three mitochondrial DNA (mtDNA) mutations (m.3460G>A in the *MT-ND1* gene, m.11778G>A in the *MT-ND4* gene, and m.14484T>C in the *MT-ND6* gene) are the main etiological factors for more than 95% of LHON cases [3], whereas other rare mutations and/or unclear factors may account for the remaining 5%. We and others recently reported several (provisional) pathogenic mutations (e.g., mutations m.3635G>A and m.10680G>A) in patients who present typical clinical characteristics of LHON but lack a reported pathogenic mutation [4-9]. The two clinical features of LHON, incomplete penetrance and male-biased onset, suggest that additional genetic or environmental factors could influence the expression of this disease [10,11]. Secondary mtDNA mutations with little deleterious effect on mitochondrial functions, some of which are haplogroup-specific variants, have been demonstrated to be influential factors for clinical expression of LHON [1,3,12]. Indeed, recent studies have found that mtDNA backgrounds could contribute to the increased or decreased penetrance of LHON [13-15].

Mutation m.3635G>A was first identified in a Russian family with LHON [4], and was considered to affect mitochondrial complex I-dependent oxygen utilization. This mutation changes a highly conserved serine to asparagine at the 110th residue of the MT-ND1 protein. In recent years, mutation m.3635G>A was occasionally reported in various Chinese families and/or singleton cases with LHON [5,7,8]. The frequency of m.3635G>A was found to be similar to that of m.3460G>A in Chinese patients with LHON [8] and was regarded as a primary LHON mutation for Chinese [7]. However, no comprehensive analysis of the mtDNA genome sequence variation in these patients with LHON and m.3635G>A has been conducted to date.

In this study, we investigated the entire mtDNA genomes of five Chinese patients with LHON and m.3635G>A. We analyzed the new sequence data, together with four previously reported mtDNA sequences harboring m.3635G>A by using a phylogenetic approach. We aimed to learn the origin of m.3635G>A in Han Chinese and to identify potentially functional variants that may act in synergy with m.3635G>A. Our results suggested multiple origins of m.3635G>A in Han Chinese.

## Methods

### Samples

Five Chinese patients (Le131, Le329, Le337, Le834, and Le569) were recruited at the Genetic Clinic of the Eye Hospital, Zhongshan Ophthalmic Center with written informed consent from all subjects. These patients received ophthalmological examinations at the Zhongshan Ophthalmic Center and were diagnosed as LHON. Patients Le131 (male; age 13 at onset), Le329 (male; age 15 at onset) and Le834 (female; age 15 at onset) had a family history of LHON, and patients Le337 (male; age 33 at onset) and Le569 (male; age 15 at onset) were sporadic. The clinical information has been reported elsewhere [8]. Patient peripheral blood samples were collected in vacuum tubes containing EDTA and were preserved at -40 °C prior to use. Genomic DNA was isolated by using a standard phenol/chloroform method. This study was approved by the institutional review board of the Kunming Institute of Zoology.

### Entire mitochondrial DNA sequence analysis

The complete mtDNA sequence was amplified and sequenced using our previously described method [16,17]. In brief, the entire mtDNA genome was amplified by using nine pair of primers on the GeneAmp PCR System 9700 (Applied Biosystems, Foster City, CA; [16,17] for detailed primers and PCR procedure). Purified PCR products were directly sequenced on a 3730 DNA sequencer (Applied Biosystems) by using BigDye Terminator v3.1 Cycle Sequencing Kit (Applied Biosystems) and 66 inner sequencing primers ([17] for primer information) following the manufacturer’s manual. Sequences were handled with the DNASTAR program (DNAS Inc., Madison, WI), and mutations/variants were scored relative to the revised Cambridge Reference Sequence (rCRS) [18]. Phylogenetic analysis of the five mtDNA sequences (Le131, Le329, Le337, Le834, and Le569) in this study, plus another four previously reported mtDNA sequences (Le1143, EU807741.1, FJ969382.1, and FJ969383.1) with m.3635G>A [4,5,7], was conducted following the same approach as in our recent studies [14,19,20]. Briefly, the haplogroup status of each mtDNA was classified according to the updated East Asian mtDNA tree and Phylotree (mtDNA tree Build 14, 5 Apr 2012) [21-23] and was validated with MitoTool [24]. To show the relationship among these mtDNAs and to distinguish private variants from haplogroup-specific variants, genetic variants in each mtDNA sequence were displayed in an mtDNA phylogenetic tree.

### Private variant analysis

Based on the principle that potentially pathogenic mtDNA mutations are most likely to be private mutations located in the terminal branches of phylogenetic tree [25], we analyzed potentially functional private variants (variants that are non-synonymous or located in the mitochondrial ribosomal RNA and mitochondrial tRNA genes) using the following strategies: 1) the uniqueness of the mtDNA variant was defined with an exhaustive database search according to the available guidelines [26]; 2) variant frequency was calculated as the number of occurrences of the variant in 15,859 complete or nearly complete mtDNA sequences summarized by the MitoTool database [24]; 3) the evolutionary conservation index of each variant was analyzed using the webserver MitoTool [24]; 4) the pathogenicity score of each non-synonymous variant was consulted according to the Supplemental Material by Pereira et al. [27]; and 5) the alteration in the structure of the protein transmembrane region caused by each non-synonymous variant was evaluated by using the TMpred program.

## Results and Discussion

Though mutation m.3635G>A has been reported in several families with LHON and has been recognized as a primary mutation for LHON in recent years [4,5,7,8], a comprehensive analysis of the mtDNA genome sequence variation in patients with LHON and m.3635G>A is needed to identify mtDNA mutations that enact a synergistic effect with m.3635G>A. In this study, five entire mtDNA genomes that had been reported to harbor m.3635G>A [8] were sequenced and were analyzed together with four previously reported sequences [4,5,7], to investigate the existence of private mutations of potential pathogenicity in each mtDNA.

**Figure 1:**
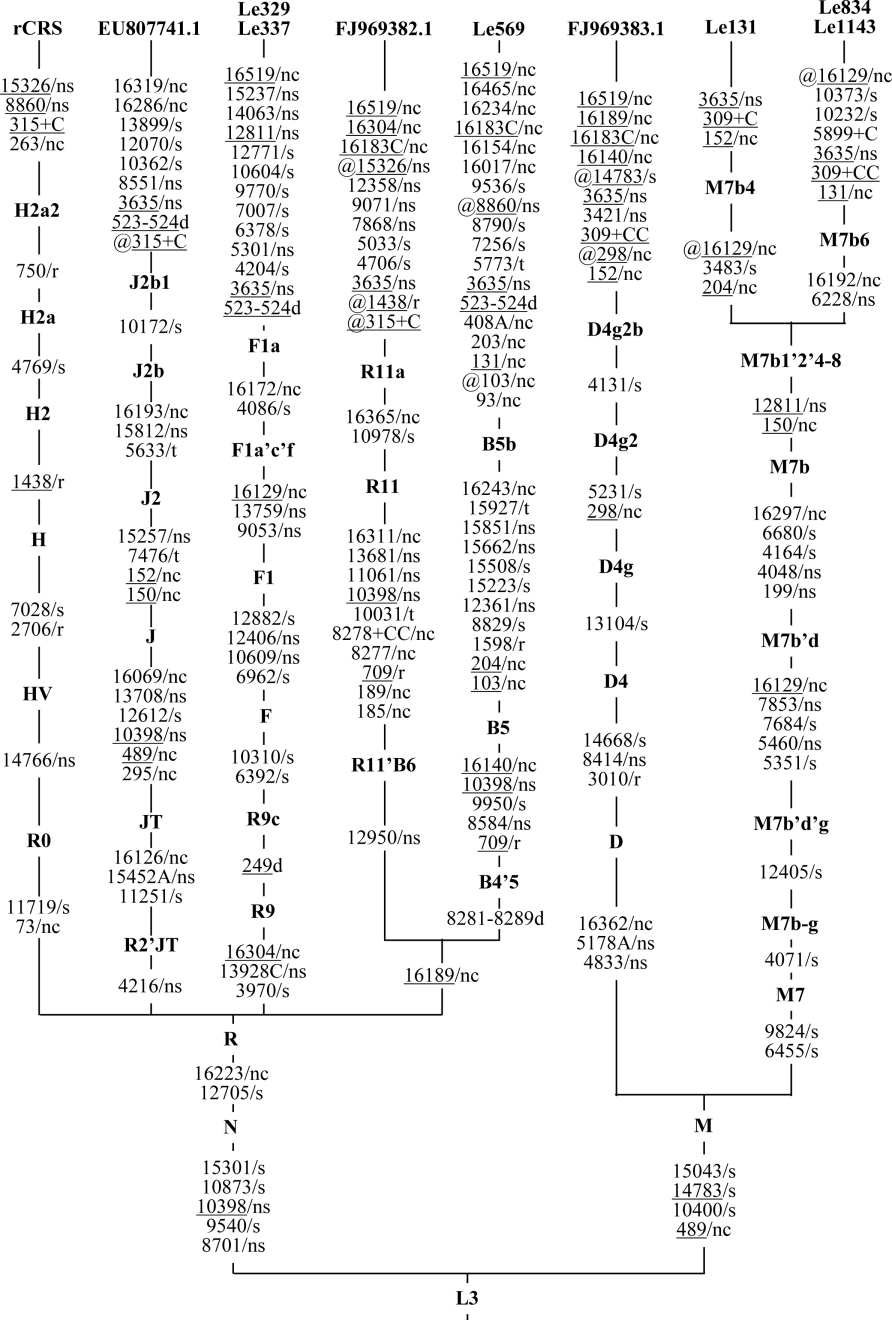
Haplogroup classification tree of nine complete mtDNA sequences with m.3635G>A. The revised Cambridge Reference Sequence (rCRS) [18] was included in the tree to show the phylogenetic position of each lineage. Deletions and insertions are denoted with a “d” and “+”, respectively; “r” indicates the variant occurs in the rRNA genes; “t” indicates the variant occurs in the tRNA genes; “nc” indicates the variant occurs in the non-coding region; synonymous and non-synonymous variants are labeled “s” and “ns”, respectively; suffixes A, C, and G mean transversions; recurrent mutations are underlined; back mutations are underlined and marked “@”.

The entire mtDNA genome sequences of patients Le131, Le329, Le337, Le569, and Le834 in this study (sequences were deposited in GenBank under Accession number JX024564-JX024568), and four other previously reported sequences with m.3635G>A (Le1143, EU807741.1, FJ969382.1, and FJ969383.1) [4,5,7], were analyzed by using a phylogenetic method. Sequence variations in each mtDNA were displayed in a phylogenetic tree (Figure 1). According to the updated East Asian mtDNA tree and Phylotree (mtDNA tree Build 14, 5 Apr 2012) [21-23], the five sequences generated in this study can be classified into haplogroups M7b4 (Le131), F1a (Le329 and Le337), B5b (Le569), and M7b6 (Le834), whereas the haplogroup status of previously reported sequences is J2b1 (EU807741.1), R11a (FJ969382.1), D4g2b (FJ969383.1), and M7b6 (Le1143). Note that the mtDNA genome sequences were identical for Le329 and Le337, Le834 and Le1143, which implies that these samples may share recent common maternal ancestors. Most of these sequences showed different haplogroup status, indicating multiple origins of m.3635G>A, similar to the other mtDNA pathogenic mutations [14,15,21,28]. We further searched the occurrence of m.3635G>A in 15,859 complete or nearly complete mtDNA sequences from worldwide populations summarized by the MitoTool web server [24]. No other sequence containing m.3635G>A was identified (Table 1).

**Table 1:** The nonsynonymous and tRNA private variants in complete mtDNA sequences with m.3635G>A.

Sample^a^	Private variant (amino acid change)	Gene	Reported (population context)^b^	Reported (disease context)^b^	Haplogroup-specific variant^c^	Variant frequency^d^	Conservation Index (CI)^e^	Pathogenic score^f^
All^g^	m.3635G>A (p.S110N)	MT-ND1	No	Yes	No	4/15859	1	0.797
Le329, Le337	m.5301A>G (p.I278V)	MT-ND2	Yes	Yes	Yes (M6, D5, H1e1a1)	125/15859	0.75	0.311
	m.14063T>C (p.I576T)	MT-ND5	Yes	Yes	No	12/15859	0.25	0.582
	m.12811T>C (p.Y159H)	MT-ND5	Yes	Yes	Yes (H3h, A2h1, M7b1’2’4–8)	124/15859	0.58	0.587
	m.15237T>C (p.I164T)	MT-CYB	Yes	No	No	2/15859	1	0.508
Le569	m.5773G>A	MT-TC	Yes	Yes	Yes (L0d3, A5a1a1, M13a, M24, M39a, L3b, D4i1, J1c1b1, H4a1b, K1a2a, C1c1b, H4a1a3)	220/15859	0.25	
EU807741.1	m.8551T>C (p.F9L)	MT-ATP6	Yes	Yes	No	10/15859	1	0.745
FJ969382.1	m.7868C>T (p.L95F)	MT-CO2	Yes	Yes	No	12/15859	0.15	0.539
	m.9071C>T (p.S182L)	MT-ATP6	Yes	Yes	Yes (H16c)	5/15859	0.65	0.223
	m.12358A>G (p.T8A)	MT-ND5	Yes	Yes	Yes (B4e, P4a, N1a1b, M7d, N9a, U5a1b1d, M12a, M27, D4b2b2, D4j1a)	216/15859	0.77	0.265
FJ969383.1	m.3421G>A (p.V39I)	MT-ND1	Yes	Yes	Yes (D4n, HV1a3, W3a1a1, H81a)	32/15859	0.37	0.413

^a^The complete mtDNA genomes of Le131, Le834 and Le1143 contained no private non-synonymous and mt-tRNA variants and was not included in the table. ^b^The uniqueness of each variant was searched according to the described strategy [26] on 10 August, 2012 (e.g., both “G3635A mtDNA” and “3635G>A mtDNA” were queried). ^c^The haplogroup-specific variant was determined according to the available global mtDNA phylogenetic tree at the Phylotree (mtDNA tree Build 14, 5 Apr 2012). The haplogroup status as it defined in that tree was indicated in round brackets. ^d^ The variant frequency was calculated as the number of occurrences of each variant in 15,859 complete or near complete mtDNA sequences that were summarized by MitoTool project [24]. ^e^The conservation index which was defined by Ruiz-Pesini et al. [30], was calculated by using MitoTool [24] concerning 43 primate species. A CI value of 0.419 meant 41.9% of 43 primate species share the same allele with the revised Cambridge Reference Sequence (rCRS) [18] (GenBank accession number NC_012920). ^f^The pathogenicity score was referred to the recent study of Pereira et al. [27]. The score ranges from 0 to 1. Higher pathogenicity score is associated with greater possibility that the variant is pathogenic. ^g^All samples contain m.3635G>A.

**Figure 2:**
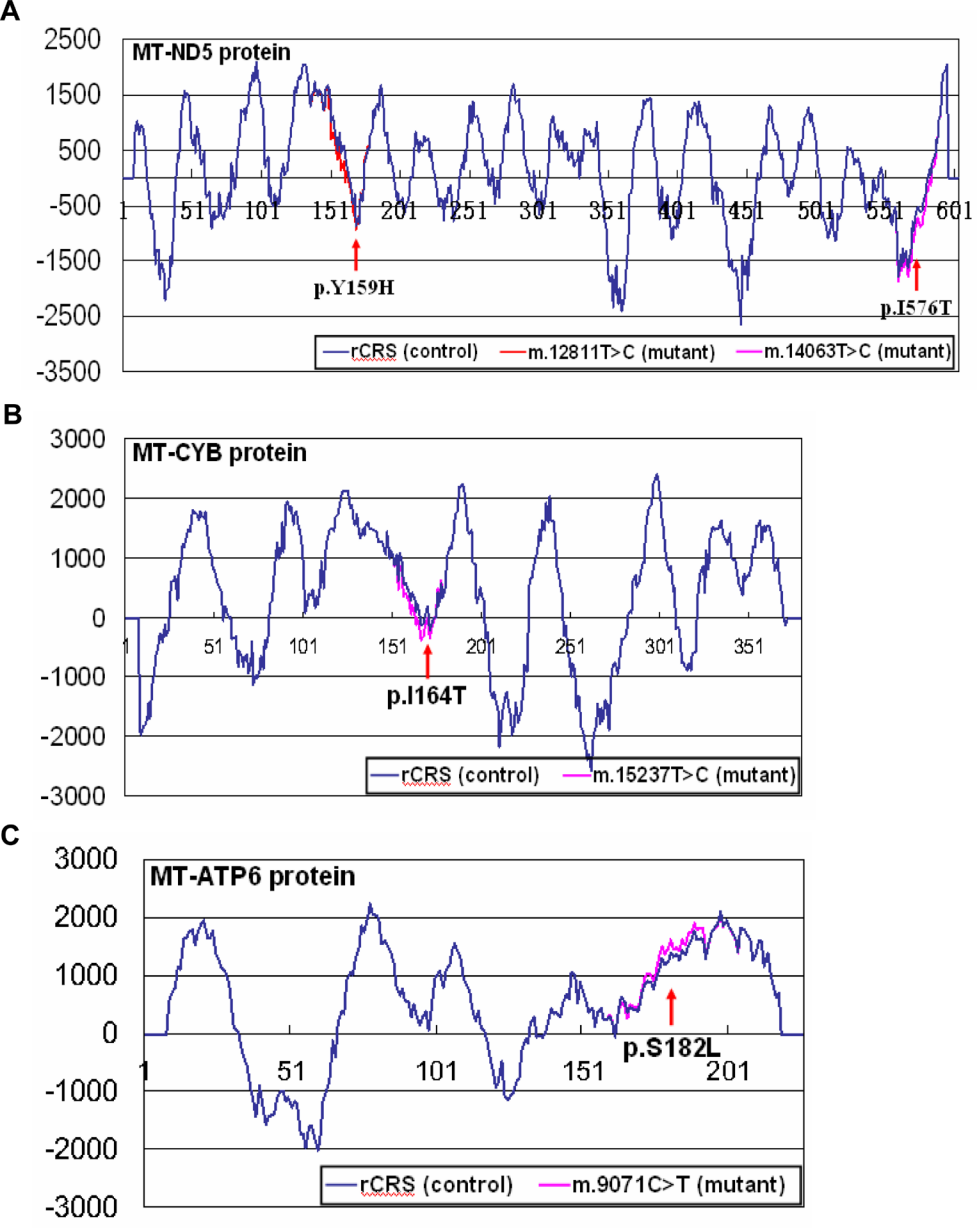
Membrane-spanning region prediction plot produced by the TMpred program. The protein membrane-spanning regions and their orientation were predicted by the TMPRED program. **A**: Variants m.12811T>C (p.Y159H) and m.14063T>C (p.I576T) changed the structure of the membrane-spanning region of the MT-ND5 protein. **B**: Variant m.15237T>C (p.I164T) changed the structure of the membrane-spanning region of the MT-CYB protein. **C**: Variant m.9071C>T (p.S182L) changed the structure of the membrane-spanning region of the MT-ATP6 protein.

Previous studies have shown that some mtDNA variants could play a synergistic role with the primary mutation affecting the clinical expression of LHON [6,12]. To explore whether potentially functional mutations could act in synergy with m.3635G>A, the non-synonymous, mitochondrial tRNA and mitochondrial ribosomal RNA private variants of each sequence were evaluated for uniqueness, frequency, conservation, pathogenicity, and potential ability to change the structure of protein transmembrane regions (Table 1 and Figure 2). Except m.3635G>A, a total of ten non-synonymous and mt tRNA private variants were identified in Le329 and Le337 (m.5301A>G [MT-ND2: p.I278V]; m.14063T>C [MT-ND5: p.I576T]; m.12811T>C [MT-ND5: p.Y159H] and m.15237T>C [MT-CYB: p.I164T]), Le569 (m.5773G>A, [MT-TC]), EU807741.1 (m.8551T>C [MT-ATP6: p.F9L]), FJ969382.1 (m.7868C>T [MT-CO2: p.L95F]; m.9071C>T [MT-ATP6: p.S182L] and m.12358A>G [MT-ND5: p.T8A]), and FJ969383.1 (m.3421G>A [MT-ND1: p.V39I]; Table 1). Employing standard database and web-based searches, we found all of these variants had been previously reported in general populations (Table 1); thus, these variants should be best regarded as polymorphisms.

None of these mtDNA variants was reported to be a “confirmed” or “suspected” LHON-associated mutation, except m.12811T>C in family Le329 and sample Le337, which was regarded as a secondary mutation for LHON [29] and was considered the main reason for the association between haplogroup M7b1’2 and the increased risk of LHON expression in our previous study [14]. Further analysis of private variants in samples Le329 and Le337 revealed another two potentially functional non-synonymous variants (m.14063T>C and m.15237T>C, Table 1, Figure 1). These two variants, together with m.12811T>C, showed the ability to change the structure of the protein membrane-spanning region (Table 1, Figure 2A-B). However, sample Le337 was sporadic, and family Le329 was too small to estimate the function of these variants on LHON penetrance [8]. Further experimental assays should be performed to characterize the function of these variants.

Among all LHON cases with m.3635G>A analyzed in this study, families EU807741.1 (family E in [4]) and FJ969382.1 (family LHON-001 in [5]) showed a relatively higher LHON penetrance. Sequence EU807741.1 [4] contained one non-synonymous private variant m.8551T>C, but it did not change the MT-ATP6 protein transmembrane structure (data not shown). Functional assessment of this mutation in a previous study also failed to support the mutation’s pathogenicity [4]. However, the potentially deleterious effect of this variant could not be fully excluded because of its low frequency, high conservation index, and high pathogenic score (Table 1). Mutation m.7868C>T was considered the reason for the high penetrance of LHON in family LHON-001 [5]. Our reanalysis of this sequence revealed another potentially functional non-synonymous variant m.9071C>T in this lineage, with the ability to influence the membrane-spanning region structure (Figure 2C), indicating its potentially synergic role with m.3635G>A.

Note that some of the private variants in these patients are haplogroup-specific variations for other haplogroups (for details, please refer to the updated East Asian mtDNA tree and Phylotree; mtDNA tree Build 14, 5 Apr 2012 [21-23]): e.g., m.5301A>G and m.12811T>C in sequences Le329 and Le337 are haplogroup-specific variants for haplogroups D5 and M7b1’2’4–8, respectively; m.5773G>A in sequence Le569 is a haplogroup-specific variant for many haplogroups, including A5a1a1, L0d3, and others; m.9071C>T and m.12358A>G in sequence FJ969382.1 are haplogroup-specific variants for haplogroups H16c and B4e, respectively; and m.3421G>A in sequence FJ969383.1 is a haplogroup-specific variant for multiple haplogroups such as D4n, HV1a3, and so on (Table 1). Except for variants m.12811T>C and m.9071C>T that have been discussed above, these variants widely distributed in general populations, and thus are unlikely to be pathogenic. Furthermore, no alteration in the protein transmembrane structure was observed for proteins with these variants (data not shown), further excluding their potentially synergic role with m.3635G>A. Through analyzing all private variants in each sequence, no additional potentially deleterious mtDNA mutation was found in patients Le131, Le569, Le834, Le1143 [7], and FJ969383.1 [5] (Table 1).

To sum up, the complete mtDNA genome sequence variation in Chinese patients with m.3635G>A was comprehensively analyzed in this study. Our results showed that m.3635G>A arose independently in different mtDNA haplogroups in Chinese families. Through analyzing mtDNA private variants in patients with LHON and m.3635G>A, we identified several variants that may act in synergy with m.3635G>A. However, we used only the evolutionary conservation analysis and predicted alteration of the protein transmembrane structure to assess the potential pathogenicity of a mutation, which may not be sufficient to make a firm conclusion. Further genetic and functional studies are essential to characterize the role of these variants in the pathogenesis of LHON.
